# LncRNA H19 regulates PI3K–Akt signal pathway by functioning as a ceRNA and predicts poor prognosis in colorectal cancer: integrative analysis of dysregulated ncRNA-associated ceRNA network

**DOI:** 10.1186/s12935-019-0866-2

**Published:** 2019-05-30

**Authors:** Min-Er Zhong, Yanyu Chen, Guannan Zhang, Lai Xu, Wei Ge, Bin Wu

**Affiliations:** 1 Department of General Surgery, Peking Union Medical College Hospital, Chinese Academy of Medical Sciences and Peking Union Medical College, No. 1 Shuaifuyuan Road, Wangfujing, Dongcheng District, Beijing, 100730 China; 2 National Key Laboratory of Medical Molecular Biology & Department of Immunology, Institute of Basic Medical Sciences, Chinese Academy of Medical Sciences, 5 Dong Dan San Tiao, Dongcheng District, Beijing, 100005 China

**Keywords:** Colorectal cancer, Competing endogenous RNA network, H19, Long non-coding RNA, TCGA

## Abstract

**Background:**

It is becoming increasingly clear that cancers can rarely be ascribed to just one or a few genomic variations. Genes generally do not function alone, but in groups that function as “networks”. This study aimed to develop a competing endogenous RNA (ceRNA) network to elucidate the role of long non-coding RNA H19 in colorectal cancer.

**Methods:**

Large-scale RNA-seq data was obtained from The Cancer Genome Atlas database. Differentially expressed RNAs were identified by bioinformatics analysis, and a competing endogenous RNA network was constructed. Functional enrichment analysis and correlation analysis between competing endogenous RNAs and clinical features were performed to reveal their roles in the tumorigenesis of colorectal cancer. To verify the conclusions derived from bioinformatics analysis, we investigated the effect of lncRNA H19 knockdown in human colorectal cancer cell lines HT-29 and HCT116.

**Results:**

The present study successfully identify various cancer-specific lncRNAs and pseudogenes in CRC. The lncRNA/pseudogene–miRNA–mRNA ceRNA network was constructed using 10 lncRNAs, 5 pseudogenes, 122 mRNAs and 39 miRNAs. In the ceRNA network of CRC, H19 up-regulates various cancer-related mRNA by competitively sponging various miRNA, and participates in PI3K–Akt signaling pathway in this manner. Cox regression and correlation analysis showed that H19 and some other competing endogenous RNAs in the network are associated with poor prognosis and clinical parameters such as tumor grade and metastasis. Knockdown of H19 reduces the protein level of MET, ZEB1, and COL1A1 in vitro.

**Conclusions:**

H19 regulates PI3K–Akt signal pathway through a competing endogenous RNA network and predicts poor prognosis in colorectal cancer. The pseudogene RPLP0P2 may be an important oncogene like H19 and needs to be studied further.

**Electronic supplementary material:**

The online version of this article (10.1186/s12935-019-0866-2) contains supplementary material, which is available to authorized users.

## Background

Colorectal cancer (CRC) ranks as the third most common malignancy in the world and is the fourth leading cause of cancer-related death. There were an estimated 1.4 million new cases of CRC and almost 700,000 deaths due to the disease in 2012 alone [1]. The onset and progression of CRC is a complex process involving many factors, and the molecular events responsible for the poor prognosis of CRC remain obscure. Understanding the molecular processes of CRC carcinogenesis is pivotal for improving early diagnosis, predicting prognosis, and developing effective therapies.

Rapid advances in genome-wide sequencing have helped researchers clarify the pathological mechanisms underlying various cancers. Aberrant transcriptomes are common in cancer. They result in abnormal production of protein-coding mRNAs and deregulated expression of the non-coding region of the genome. Non-coding RNAs (ncRNAs) have coding-independent functions in the regulation of important biological processes such as cell development, differentiation, and proliferation.

Long non-coding RNAs (lncRNA) refer to transcripts that are > 200 nucleotides long and are not translated into protein. A large number of lncRNAs have been identified in various types of cancer. They regulate complex cellular behaviors that are commonly deregulated in cancer (e.g., growth, differentiation, and establishment of cell identity). Some lncRNAs have been demonstrated to be associated with poor prognosis in various cancers and are now used as biomarkers.

LncRNA H19 has been reported to have key regulatory functions in tumor development and progression. Recently, H19 has been shown to be involved in opposed processes, e.g., in cell proliferation and differentiation, as well as in epithelial–mesenchymal transition (EMT) and mesenchymal–epithelial transition. Nevertheless, currently available evidence supports the oncogenic properties of H19. The competing endogenous RNA (ceRNA) hypothesis is new theory that helps explain the intrinsic mechanisms of ncRNA. MicroRNAs (miRNAs) post-transcriptionally regulate gene expression by binding to specific recognition sites known as miRNA response elements (MREs) on target transcripts. The ceRNA hypothesis postulates that lncRNAs, pseudogenes, and other RNA transcripts that harbor MREs could act as endogenous miRNA sponges and inhibit miRNA function, and thereby impact the targets of multiple miRNAs [2]. A previous study has shown that H19 promotes cell migration and invasion in cholangiocarcinoma by functioning as a ceRNA [3]. H19 may also play an oncogenic role in human CRC. Silencing H19 expression has been shown to cause a noticeable reduction in cancer cell proliferation and migration [4]. These findings stimulated our interest in investigating the role of H19 in the ceRNA regulatory network in CRC progression.

In the present study, we constructed a CRC-specific ceRNA network using a large cohort from The Cancer Genome Atlas (TCGA) database and attempted to elucidate the post-transcriptional regulator role of H19. Moreover, we aimed to use the ceRNA network to help with in-depth study of the lncRNA/pseudogene–miRNA–mRNA crosstalk in CRC and thus obtain insights into the molecular mechanisms involved in the tumorigenesis and progression of CRC.

## Methods

### Patients and TCGA data retrieval

The RNA sequence data of CRC patients and the corresponding clinical information were obtained from the TCGA data portal (https://portal.gdc.cancer.gov/). According to the TCGA project’s large-scale study of CRC specimens, the pattern of genomic alterations in CRC tissue is the same regardless of whether tumor origin is in the colon or the rectum, leading to the conclusion that these two cancer types can be grouped as one [5]. Therefore, for this analysis, we merged the datasets of colon adenocarcinoma and rectum adenocarcinoma.

This study is in accordance with the publication guidelines provided by TCGA. The RNA profiles data and the clinical characteristics of colon adenocarcinoma and rectum adenocarcinoma are publicly available in open-access platforms, therefore approval by the local ethics committee was not needed.

### RNA sequence data processing

The RNA expression data (level 3) of CRC patients—obtained from 622 CRC cancer tissues and 51 adjacent non-tumor normal tissues (up to June 13, 2018)—were downloaded from the TCGA data portal. The expression profiles of RNA and miRNA from the 673 samples had been derived from the IlluminaHiSeq RNASeq and the IlluminaHiSeq miRNASeq sequencing platforms. The mRNAs, lncRNAs, and pseudogenes were identified based on the annotation from the Ensembl database (http://www.ensembl.org/index.html, version 93). RNAs not included in the Ensembl database were excluded from the present study. We mainly used the R program (R Foundation for Statistical Computing, Vienna, Austria. URL http://www.R-project.org/) for analysis of RNA data. Raw counts data were normalized by the edgeR package [6] and then transformed by the limma package [7].

### Identification of differentially expressed mRNAs, lncRNAs, pseudogenes, and miRNAs

The differential expression of mRNAs, lncRNAs, pseudogenes, and miRNAs between CRC and adjacent normal tissue were identified individually by using the limma package in R. False discovery rate (FDR) was introduced to correct the statistical significance of the multiple test. | Log2 fold change (FC)| ≥ 1.0 and FDR < 0.01 were set as the thresholds. For the identified differentially expressed mRNAs, lncRNAs, pseudogenes, and miRNAs, we generated volcano maps using the ggplot2 package [8] in R.

### CeRNA network construction

Interactions between differentially expressed lncRNAs and miRNAs, as well as interactions between differentially expressed pseudogenes and miRNAs, were predicted using the starBase database (http://starbase.sysu.edu.cn) [9]. The starBase database is designed for decoding the interaction networks of lncRNAs, miRNAs, ceRNAs, RNA-binding proteins, and mRNAs from large-scale crosslinking-immunoprecipitation and high-throughput sequencing (CLIP-seq) data and tumor samples. The mRNAs targeted by the differentially expressed miRNAs were also retrieved using the starBase database. Then, the GDCRNATools package [10] in R was introduced to construct the ceRNA regulatory network. The GDCRNATools package uses three criteria to identify competing lncRNA–mRNA or pseudogene–mRNA pairs: (1) the number and hypergeometric probability of shared miRNAs by a lncRNA/pseudogene–mRNA pair, (2) the strength of positive expression correlation between lncRNA/pseudogene and mRNA, and (3) the overall regulation similarity of all shared miRNAs on the lncRNA/pseudogene–mRNA pair. Finally, a lncRNA/pseudogene–miRNA–mRNA ceRNA network was constructed based on the differentially expressed miRNA–lncRNA, differentially expressed miRNA–pseudogene, and differentially expressed miRNA–mRNA interactions. The network was visualized using the Cytoscape 3.6.1 software. Linear regression analysis was performed to evaluate the correlation of expression levels between ceRNA pairs.

### Functional enrichment analysis

Considering that mRNAs are the implementers of molecular function in the ceRNA network, functional enrichment analysis was performed to reveal the functional implications of these mRNAs in the tumorigenesis of CRC. Both Gene Ontology (GO) functional enrichment analysis and Kyoto Encyclopedia of Genes and Genomes (KEGG) pathway enrichment analysis were conducted using the clusterProfiler package [11]. FDR < 0.05 was set as the threshold for statistical significance for both GO and KEGG enrichment analysis.

### Cell culture

Colorectal cancer cell lines HT-29 and HCT116 were obtained from China Infrastructure of Cell Line Resources (Beijing, China). HT-29 was cultured in Dulbecco’s Modified Eagle Medium: Nutrient Mixture F-12 (DMEM/F-12; Thermo Fisher Scientific, Waltham, MA, USA) containing 10% fetal bovine serum (Thermo Fisher Scientific). HCT116 was cultured in Iscove’s modified Dulbecco’s medium (IMDM; HyClone, Logan, UT, USA) containing 10% fetal bovine serum. All cell cultures were maintained at 37 °C in a humidified 5% CO_2_ atmosphere. Cells were passaged approximately every 2–3 days.

### Transfection of lncRNA smart silencer

LncRNA Smart Silencer (RiboBio, Guangzhou, China) was used to knock down the expression of lncRNA H19. H19 Smart Silencer is a mixture of three siRNAs and three antisense oligonucleotides (ASOs). The target sequences of siRNAs are as follows: 5′-CGTGACAAGCAGGACATGA-3′, 5′-CCCACAACATGAAAGAAAT-3′, 5′-GACGTGACAAGCAGGACAT-3′. The target sequences of ASOs are as follows: 5′-GGCCTTCCTGAACACCTTAG-3′, 5′-GCAGGACATGACATGGTCCG-3′, 5′-GGACGTGACAAGCAGGACAT-3′. The negative control (NC) Smart Silencer does not contain domains homologous to humans, mice, and rats. LncRNA Smart Silencer transfection was performed with Lipofectamine 2000 (Invitrogen, Carlsbad, CA, USA) according to the manufacturer’s instructions. Approximately 1 × 10^5^ HCT116 or 3 × 10^5^ HT29 cells were plated into each well of the 12-well plate at least 24 h before transfection to achieve 70% confluency. Cells were collected 48 h after transfection for RNA isolation and Western blot.

### RNA isolation, cDNA synthesis and real-time PCR

Total RNA was extracted from cell lines using TRIzol reagent (Invitrogen) according to the manufacturer’s instructions. Complementary DNA (cDNA) synthesis was conducted with 1 μg total RNA using the PrimeScript™ II 1st Strand cDNA Synthesis Kit (cat # 6210A, Takara, Japan). The primers were obtained from Sangon Biotech (Shanghai, China) and the sequences were designed as follows: for H19, the forward primer was 5′-TGCTGCACTTTACAACCACTG-3′ and the reverse primer was 5′-ATGGTGTCTTTGATGTTGGGC-3′; for GAPDH, the forward primer was 5′-AAATCAAGTGGGGCGATGCT-3′ and the reverse primer was 5′-GTGCTAAGCAGTTGGTGGTG-3′. Real-time PCR was performed with TB Green™ Premix Ex Taq™ II (cat # RR820A, Takara) on a CFX96 Real-Time PCR Detection System (Bio-Rad, California, USA), according to the following conditions: 30 s at 95 °C for initial denaturation, followed by 40 cycles of 5 s at 95 °C for denaturation, and 30 s at 60 °C for annealing and extension. RNA expression was normalized to GAPDH; relative RNA expression was calculated through the 2^−ΔΔCt^ method.

### Western blot assay

Total proteins were extracted from whole-cell lysates using RIPA lysis buffer. The cells lysates were collected, and the concentrations were measured by BCA Protein Assay Kit (Thermo Fisher Scientific). The cell lysates were then separated by SDS-PAGE and transferred to nitrocellulose membranes (Millipore, Bedford, MA, USA). Membranes were blocked with 5% non-fat dried milk in TBS-T (TBS plus 0.05% Tween-20) for 30 min and incubated with primary detection antibodies overnight at 4 °C. The membranes were washed and then incubated with horseradish peroxidase-conjugated secondary detection antibodies and enhanced chemiluminescence reagents (Thermo Fisher Scientific). Proteins were detected with antibodies against MET (cat # 8198, Cell Signaling Technology, USA), COL1A1 (cat # ab138492) and ZEB1 (cat # ab203829, Abcam, UK). Anti-human β-actin polyclonal antibody (cat # GTX124213, GeneTex, China) was used as an internal reference.

### Survival and clinical feature analysis

JMP Pro (version 13.0; SAS Institute, Cary, NC, USA) and GraphPad Prism (version 7.04; Nashville, TN, USA) were used for the statistical analyses. The unpaired *t*-test was used to determine the significance of differences between two groups. Univariate Cox proportional hazards regression analysis was carried out to identify the lncRNAs, pseudogenes, and mRNAs whose expression correlated with overall survival (OS). For each of the differentially expressed mRNAs, lncRNAs, and pseudogenes in the ceRNA network, the CRC patients were classified into either a high-expression group or a low-expression group using the first quarter, third quarter, or median expression value of the specific RNA as the cutoff. The prognosis of each group of patients was examined by Kaplan–Meier survival analysis, and the survival outcomes of the two groups were compared by the log-rank test. Finally, the cutoff value yielding the lowest log-rank *P* value was selected. We also analyzed the association between the ceRNAs and clinical parameters such as tumor grade, lymphatic invasion, venous invasion, metastasis, and TNM stage, using Student’s *t*-test. *P* < 0.05 (two-sided) was considered significant.

## Results

### LncRNA H19 is highly expressed in CRC tissues

**Fig. 1:**
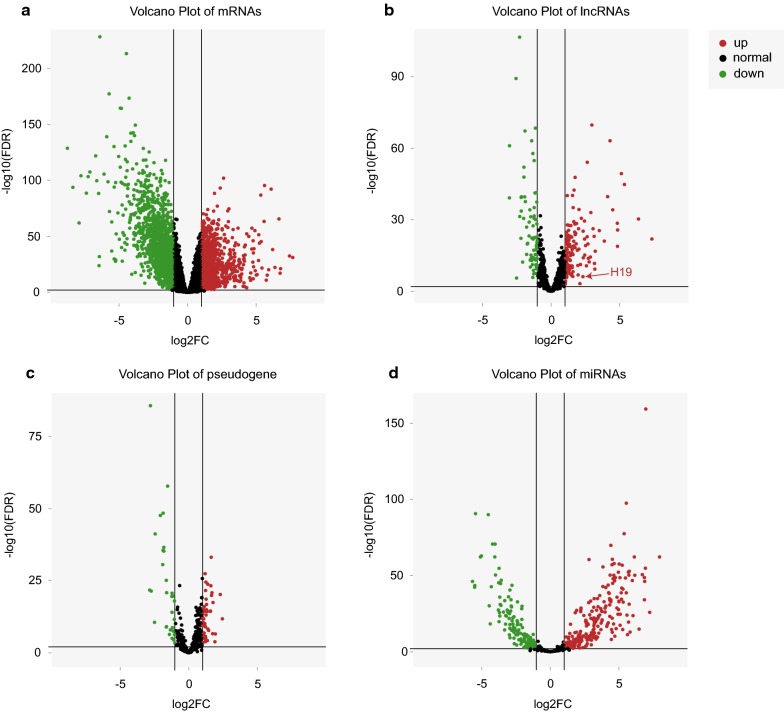
Volcano map of differentially expressed mRNAs (**a**), lncRNAs (**b**), pseudogenes (**c**), and miRNAs (**d**). The red dots in the plot represents significantly up-regulated RNAs and the green dots represents significantly down-regulated RNAs: **a** 1158 mRNAs were up-regulated, and 1816 were down-regulated; **b** 144 lncRNAs were up-regulated, and 69 were down-regulated. LncRNA H19 was found to be upregulated in human CRC primary tissues; **c** 46 pseudogenes were up-regulated, and 29 pseudogenes were down-regulated; **d** 227 miRNAs were up-regulated, and 126 were down-regulated. *FDR* false discovery rate, *FC* fold change

We detected 2974 mRNAs (Fig. 1a), 213 lncRNAs (Fig. 1b), 75 pseudogenes (Fig. 1c), and 353 miRNAs (Fig. 1d) that were differentially expressed between CRC tissues and normal colorectal tissues. In CRC tissues, 1158 mRNAs were up-regulated and 1816 were down-regulated; 144 lncRNAs were up-regulated, and 69 were down-regulated; 46 pseudogenes were up-regulated and 29 were down-regulated; and 227 miRNAs were up-regulated and 126 were down-regulated. Consistent with previous studies, H19 was found to be up-regulated in human CRC primary tissues (Fig. 1b). Figure 1 shows the distribution of all the differentially expressed genes on the two dimensions of − log10(FDR) and log2FC through volcano maps. A complete list of the differentially expressed mRNAs, lncRNAs, pseudogenes, and miRNAs is provided in Additional file 1.

### LncRNA H19 sponges 6 miRNAs and interacts with 38 mRNAs in the ceRNA network of CRC

**Fig. 2:**
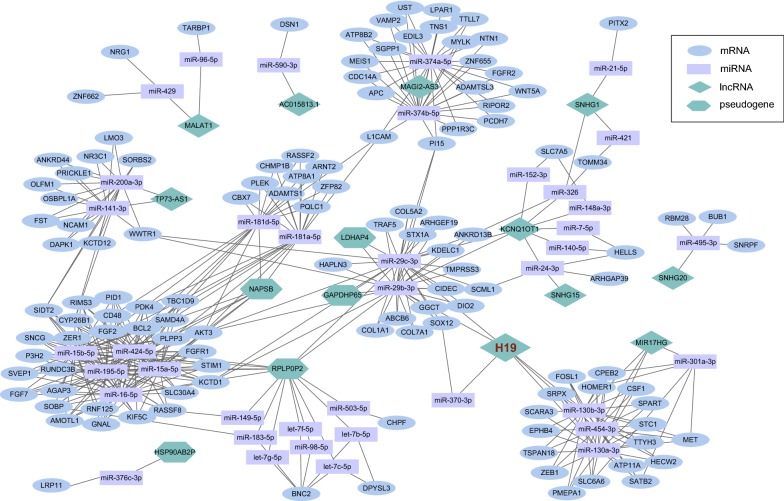
The lncRNA/pseudogene–miRNA–mRNA ceRNA network in CRC. The blue ellipses represent mRNAs, the purple rectangles represent miRNAs, the green diamonds represent lncRNAs, and and the green hexagon represents pseudogenes. There are 10 lncRNAs, 5 pseudogenes, 122 mRNAs, 39 miRNAs, and 433 interactions included in this ceRNA network. LncRNAs, pseudogenes, miRNAs, and mRNAs interact with each other through shared MREs

Combining the lncRNA/pseudogene–miRNA interactions with the miRNA–mRNA interactions, an integrated lncRNA/pseudogene–miRNA–mRNA ceRNA network was established, consisting of 176 nodes and 433 interactions (Fig. 2). There were 10 lncRNAs, 5 pseudogenes, 122 mRNAs, and 39 miRNAs in the ceRNA network. The 15 lncRNAs and pseudogenes that acted as ceRNA in the network, and their target miRNAs, are shown in Table 1. The top 10 miRNAs, with their target mRNAs, are shown in Table 2. A complete list of all 39 miRNAs, with their target mRNAs, are shown in Additional file 2.

**Table 1:** The lncRNAs/pseudogenes and their target miRNAs in the ceRNA network

Gene	Type	Log_2_FC	P value	miRNAs
RPLP0P2	Pseudogene	1.77	1.42E−18^a^	miR-98-5p, miR-503-5p, miR-424-5p, miR-29c-3p, miR-29b-3p, miR-195-5p, miR-183-5p, miR-16-5p, miR-15b-5p, miR-15a-5p, miR-149-5p, let-7 g-5p, let-7f-5p, let-7c-5p, let-7b-5p
KCNQ1OT1	lncRNA	1.53	5.00E−17^a^	miR-7-5p, miR-326, miR-29c-3p, miR-29b-3p, miR-24-3p, miR-152-3p, miR-148a-3p, miR-140-5p
NAPSB	Pseudogene	− 1.82	1.21E−36^a^	miR-424-5p, miR-195-5p, miR-181d-5p, miR-181a-5p, miR-16-5p, miR-15b-5p, miR-15a-5p
H19	lncRNA	2.31	4.57E−07^a^	miR-454-3p, miR-370-3p, miR-29c-3p, miR-29b-3p, miR-130b-3p, miR-130a-3p
MIR17HG	lncRNA	2.34	1.03E−26^a^	miR-454-3p, miR-301a-3p, miR-130b-3p, miR-130a-3p
SNHG1	lncRNA	1.75	9.53E−50^a^	miR-421, miR-326, miR-24-3p, miR-21-5p
MALAT1	lncRNA	1.15	1.47E−07^a^	miR-96-5p, miR-429
LDHAP4	Pseudogene	1.23	3.73E−06^a^	miR-29c-3p, miR-29b-3p
GAPDHP65	Pseudogene	1.19	1.27E−06^a^	miR-29c-3p, miR-29b-3p
TP73-AS1	lncRNA	− 1.87	1.07E−49^a^	miR-200a-3p, miR-141-3p
MAGI2-AS3	lncRNA	− 1.59	6.55E−30^a^	miR-374b-5p, miR-374a-5p
HSP90AB2P	Pseudogene	1.07	2.86E−07^a^	miR-376c-3p
SNHG15	lncRNA	1.75	9.53E−50^a^	miR-24-3p
SNHG20	lncRNA	1.12	4.65E−25^a^	miR-495-3p
AC015813.1	lncRNA	1.01	1.45E−09^a^	miR-590-3p

*FC* fold change

^a^Statistically significant

**Table 2:** The top 10 miRNAs targeted most mRNAs in the ceRNA network

miRNA	mRNA
miR-15a-5p/miR-424-5p/miR-16-5p/miR-195-5p^a^	PDK4, RUNDC3B, SLC30A4, KIF5C, PLPP3, BCL2, ZER1, STIM1, SNCG, CD48, RNF125, P3H2, RIMS3, GNAL, SVEP1, SIDT2, TBC1D9, FGF2, PID1, AMOTL1, CYP26B1, FGF7, SAMD4A, AKT3, FGFR1, KCTD1, AGAP3, SOBP, RASSF8
miR-15b-5p	PDK4, RUNDC3B, SLC30A4, KIF5C, PLPP3, BCL2, ZER1, STIM1, SNCG, CD48, RNF125, P3H2, RIMS3, GNAL, SVEP1, SIDT2, TBC1D9, FGF2, PID1, AMOTL1, CYP26B1, FGF7, SAMD4A, AKT3, FGFR1, KCTD1, AGAP3, SOBP
miR-374a-5p/miR-374b-5p^a^	CDC14A, NTN1, LPAR1, ADAMTSL3, EDIL3, VAMP2, FGFR2, MYLK, MEIS1, PPP1R3C, TTLL7, UST, L1CAM, TNS1, PCDH7, SGPP1, RIPOR2, APC, PI15, ATP8B2, ZNF655, WNT5A
miR-29b-3p/miR-29c-3p^a^	ANKRD13B, TRAF5, GGCT, SCML1, COL7A1, ARHGEF19, ABCB6, STX1A, PI15, COL1A1, AKT3, KDELC1, WWTR1, KCTD1, SOX12, COL5A2, DIO2, TMPRSS3, CIDEC, HAPLN3
miR-130a-3p/miR-130b-3p/miR-454-3p^a^	SLC6A6, MET, CPEB2, HOMER1, SATB2, SRPX, ATP11A, TTYH3, EPHB4, SCARA3, SPART, ZEB1, FOSL1, CSF1, STC1, PMEPA1, TSPAN18, HECW2
miR-181a-5p/miR-181d-5p^a^	PDK4, CBX7, PLPP3, BCL2, CHMP1B, ADAMTS1, L1CAM, TBC1D9, RASSF2, ATP8A1, PQLC1, SAMD4A, AKT3, ZFP82, ARNT2, PLEK
miR-141-3p/miR-200a-3p^a^	NCAM1, LMO3, NR3C1, OLFM1, OSBPL1A, SIDT2, ANKRD44, CYP26B1, SORBS2, KCTD12, WWTR1, DAPK1, PRICKLE1, FST
miR-301a-3p	SLC6A6, MET, CPEB2, HOMER1, SATB2
miR-24-3p	ARHGAP39, HELLS, SCML1
miR-495-3p	RBM28, BUB1, SNRPF

^a^Shared the same target mRNA in this ceRNA network

**Fig. 3:**
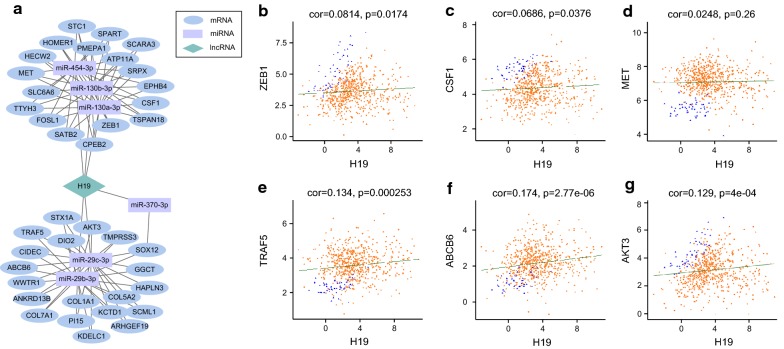
The ceRNA network of H19 and linear regression of mRNA expression levels with H19. **a** H19 regulates 6 miRNAs and interacts with 38 mRNAs in current ceRNA network. The blue ellipses represent mRNAs, the purple rectangles represent miRNAs, and the green diamonds represent lncRNAs. **b**–**g** H19 shows no significant linear correlation with all these mRNAs

Linear regression analysis showed that there was no direct linear correlation between lncRNA H19 and mRNAs in the ceRNA network. Mediated by miR-454-3p, miR-130a-3p, and miR-130b-3p, H19 interacted with ZEB1, CSF1, MET, and other 15 mRNAs. Mediated by miR-29b-3p, miR-29c-3p, and miR-370-3p, H19 interacted with TRAF5, ABCB6, AKT3, and other 17 mRNAs (Fig. 3a). However, there was no significant linear correlation of H19 with any of these mRNAs (Fig. 3b–g).

### mRNAs in the ceRNA network were enriched to the PI3K signal pathway

**Fig. 4:**
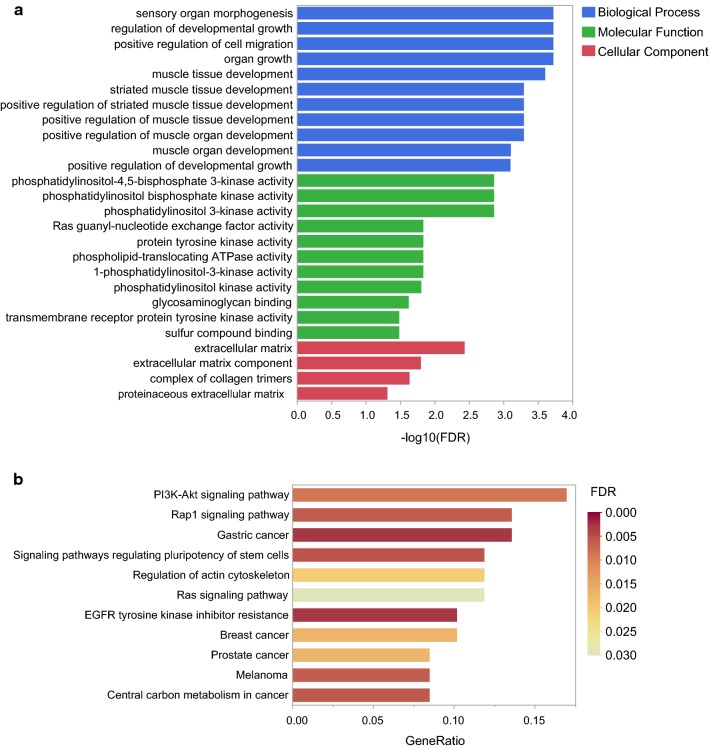
GO functional enrichment and KEGG pathway enrichment of mRNAs included in the ceRNA network. **a** Top 11 biological process terms, top 11 molecular function terms, and all cellular component terms with the most significant P values. **b** All KEGG enrichment results of mRNAs included in the ceRNA network. FDR = false discovery rate

Functional enrichment analysis revealed that a total of 259 GO terms, including 242 biological process terms, 13 molecular function terms, and 4 cellular component terms were enriched by the mRNAs in the ceRNA network. The top 11 biological process and molecular function terms, and the top 4 cellular component categories, are shown in Fig. 4a, and the complete list of all 259 GO terms are shown in Additional file 3. A total of 11 KEGG pathways related to biological pathways were enriched among the mRNAs in the ceRNA network, including the PI3K–Akt signaling pathway (hsa04151), the Ras signaling pathway (hsa04014), regulation of actin cytoskeleton (hsa04810), and the central carbon metabolism in cancer pathway (hsa05230) (Fig. 4b).

### H19 upregulation is associated with poor prognosis and other clinical parameters

**Fig. 5:**
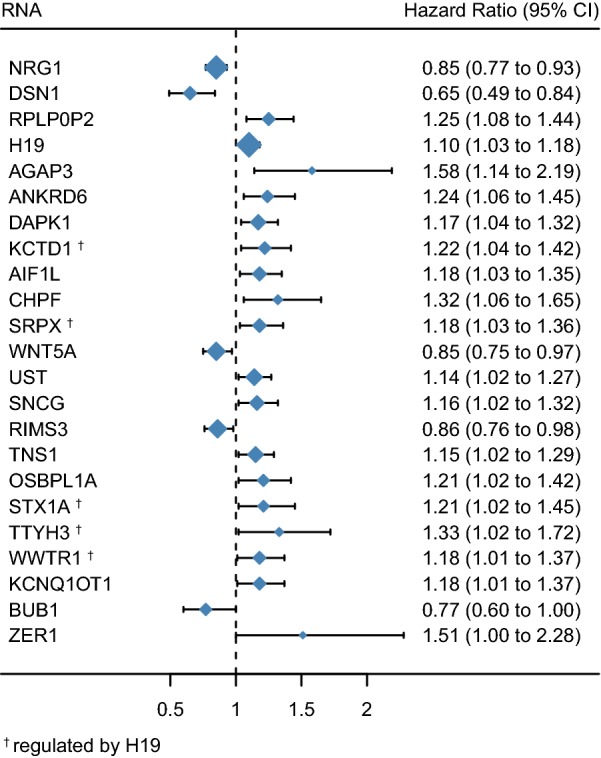
Forest plots of hazard ratios (HR) of survival associated RNAs in the ceRNA network. A total of 20 mRNAs, 2 lncRNAs, and 1 pseudogene were found to be prognostic factors. The RNAs with hazard ratio < 1 are protective factors, while the ones with hazard ratio > 1 are risk factors in CRC. The hazard ratio is the ratio of the hazard rates corresponding to the conditions described by two levels of an explanatory variable

**Fig. 6:**
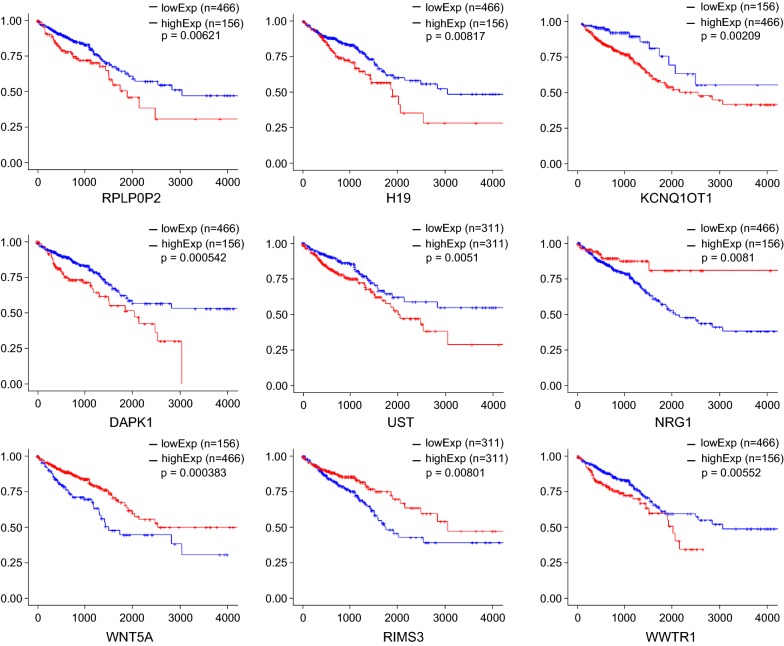
Kaplan-Meier survival curves for 1 pseudogene, 2 lncRNAs, and 6 mRNAs associated with overall survival. Horizontal axis: overall survival time (in days), Vertical axis: proportion surviving

To identify the mRNAs, lncRNAs, and pseudogenes with potential prognostic value, the expression levels of 122 mRNAs, 10 lncRNAs, and 5 pseudogenes in the ceRNA network were profiled using the univariate Cox proportional hazards regression model. A total of 20 mRNAs, 2 lncRNAs, and 1 pseudogene were identified to be prognostic factors (*P* < 0.05; Fig. 5). Five of these mRNAs were regulated by lncRNA H19 (Fig. 5). Kaplan–Meier curve analysis showed that 24 mRNAs (OSBPL1A, AGAP3, TMPRSS3, VAMP2, EPHB4, SCARA3, GNAL, TNS1, STX1A, WWTR1, ANKRD6, DAPK1, PRICKLE1, SOX12, DPYSL3, SRPX, SNCG, CHPF, TTYH3, UST, AKT3, ABCB6, TSPAN18, and SOBP) were negatively correlated with OS (*P* < 0.05). Furthermore, one pseudogene (RPLP0P2) and two lncRNAs (H19 and KCNQ1OT) were negatively correlated with OS (*P* < 0.05). Meanwhile, 14 mRNAs (NCAM1, CD48, GGCT, DIO2, PLEK, WNT5A, HOMER1, RBM28, BUB1, CPEB2, RIMS3, NCAPD3, DSN1, and NRG1) were found to be positively correlated with OS (*P* < 0.05). Figure 6 displays the Kaplan–Meier curves of one pseudogene, two lncRNAs, and six mRNAs, along with the most significant *P* values. A complete list of all RNAs correlated with OS is provided in Additional file 4.

With regard to other clinical parameters, we found that H19 upregulation is linked to tumor grade, lymphatic invasion, metastasis, and TNM stage. Apart from H19, another eight ceRNAs were associated with clinical parameters such as TNM stage and venous invasion (Table 3; *P* < 0.05).

**Table 3:** The ceRNAs associated with specific prognostic parameters

Clinical features	Upregulated	Downregulated
Tumor grade	H19, HSP90AB2P	TP73-AS1, MAGI2-AS3
Lymphatic invasion	H19, KCNQ1OT1	TP73-AS1, MAGIS-AS3
Venous invasion	HSP90AB2P	
Metastasis	H19, MIR17HR, SNHG20, KCNQ1OT1	
TNM stage	H19, SNHG15, AC015813.1	

### Knockdown of H19 reduces the protein level of MET, ZEB1, and COL1A1 in vitro

**Fig. 7:**
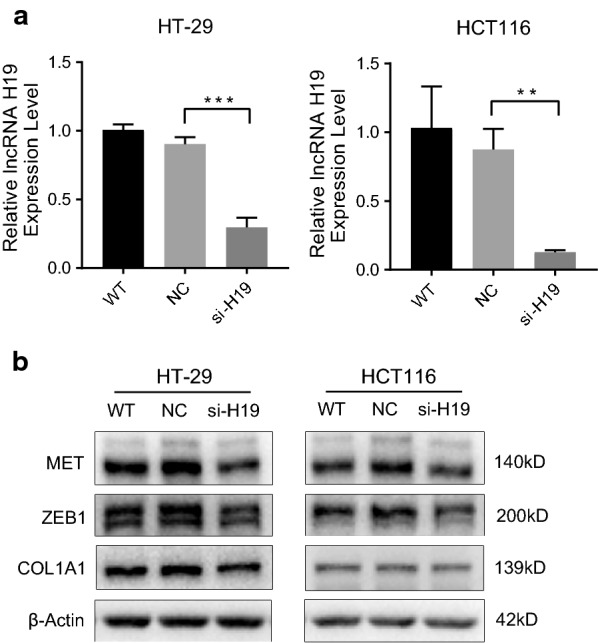
Knockdown of H19 reduces the protein level of MET, ZEB1, and COL1A1 in vitro. **a** Relative expression of lncRNA H19 was determined by real-time PCR analysis. Results are presented as the mean ± SD (**P < 0.01, ***P < 0.001). H19 knockdown was performed in human colorectal cancer cell lines HT-29 and HCT116. **b** Western blot analysis demonstrated that knockdown of H19 could downregulate the expression of MET, ZEB1, and COL1A1 in both HT-29 and HCT116 cells at the level of protein. *WT* wild type, *NC* negative control, *si-H19* cell line transfected with lncRNA H19 Smart Silencer

To verify the conclusions derived from bioinformatics analysis, we investigated how knockdown of the lncRNA H19 in CRC cell lines HT-29 and HCT116 affected protein expression. The relative expression levels indicated that lncRNA H19 was knocked down in both HT-29 and HCT116 (*P *< 0.05, Fig. 7a). Based on the current ceRNA network, we chose three proteins (MET, ZEB1, and COL1A1) that interacted with H19 for Western blot analysis. Our data demonstrated that knockdown of H19 could downregulate the expression of MET, ZEB1, and COL1A1 in both HT-29 and HCT116 cells (Fig. 7b). These results were consistent with the ceRNA network mentioned above.

## Discussion

Although over the past several decades there has been a slight decline in CRC incidence and associated mortality, it remains an important contributor to cancer incidence and mortality worldwide. A large number of CRC patients are diagnosed with advanced stage disease and have poor prognosis [12]. Therefore, increasing attention is being given to the identification of genes involved in CRC development and progress, and the exact regulatory mechanisms. Thousands of genes have already been demonstrated to play important roles in various cancer processes. Notably, lncRNA H19 is frequently over-expressed in the majority of human cancers including CRC. However, it is becoming increasingly clear that cancers can rarely be ascribed to just one or a few genomic variations. Genes generally do not function alone, but in groups that function as “networks”. A novel hypothesis—called the ceRNA hypothesis—proposes that different RNA transcripts could interact with one another through shared MREs. This new theory of gene regulation at the post-transcriptional level may help us better understand the mechanisms of various diseases, including cancers. In-depth ceRNA analysis can clarify the functions of coding and non-coding RNAs. Therefore, to elucidate the role of H19 in CRC progression, we constructed a ceRNA network based on the TCGA dataset and large-scale CLIP-Seq data. To the best of our knowledge, this is the first study on CRC to report a ceRNA network containing pseudogenes as well as lncRNAs.

Many studies have reported that lncRNAs and pseudogenes may function as ceRNA regulators to communicate with other RNA transcripts. Each ceRNA contains MREs for a combination of different miRNAs, and thus they can impact multiple targets of multiple miRNAs. One miRNA may have more than one target RNA, which enables all transcripts (including pseudogenes and lncRNA transcripts) bearing MREs to connect through the “ceRNA–miRNA–ceRNA–miRNA–” chain to form a network (Fig. 2). The ceRNA networks are intricate, and most of them are not simply linear. For instance, in the present study, mediated by 6 miRNAs, H19 interacted with 38 mRNAs (Fig. 3a). However, H19 showed no significant linear correlation with any of these mRNAs (Fig. 3b–g). Although H19 sponges 6 miRNAs and regulates 38 mRNAs, it is just a small part of the entire ceRNA network in CRC. The ceRNA network had complex combinations in terms of competition. For example, the cancer-related gene *WWTR1* was coregulated by H19 and lncRNA TP73-AS1 through four different miRNAs (Fig. 2). Alterations in one ceRNA might have major effects on the entire ceRNA network. Indirect ceRNA interactions amplify ceRNA influence in gene regulation. Thus, in both physiological and pathological conditions, there is functional complexity, diversification, and built-in regulatory loops. According to Salmena et al. [2] the most robust ceRNA networks will include transcripts that share multiple MREs targeted by multiple miRNAs.

The ceRNAs may play a procarcinogenic role by competitively binding miRNA to regulate mRNA expression levels. The GO enrichment results suggested that dysregulation of mRNAs results in significant alteration in cell migration, regulation of developmental growth, and molecular functions closely related to the PI3K–Akt signaling pathway (Fig. 4). Based on the KEGG pathway database, the PI3K–Akt signaling was found to involve the maximum number of differentially expressed mRNAs. The PI3K–Akt signaling pathway is well known to play an integral role in many cellular processes; it is frequently altered in cancer, and has been shown to contribute to tumor growth and survival [13]. Previous studies have demonstrated that receptor tyrosine kinases exert dominant control over PI3K signaling in human *KRAS*-mutant colorectal cancers [14]. Further, PI3K–Akt signaling cooperates with Wnt to increase beta-catenin signaling during inflammation. Beta-catenin signaling, induced by PI3K and mediated by Akt, appears to be essential for activation of progenitor cells during progression from ulcerative colitis to CRC [15, 16]. In the present study, mRNAs regulated by H19 (AKT3, CSF1, MET, COL1A1) were mainly enriched in PI3K–Akt signaling pathway. Moreover, our experimental data demonstrated that H19 could regulate the expression of MET, ZEB1, and COL1A1 in both HT-29 and HCT116 cells (Fig. 7b).

Taken together the evidence suggests that H19 up-regulates various cancer-related mRNA expression levels via serving as a ceRNA, and participates in the PI3K–Akt signaling pathway in this manner, playing a key role in promoting cancer progression. The miRNAs targeted by H19 in the current ceRNA network (Table 1) were demonstrated to play a vital role in tumorigenesis. For instance, miR-130 was identified to be an oncogenic miRNA [17]. Mutant p53 gain-of-function induces EMT through modulation of the miR-130b-ZEB1 axis [18], and this axis was regulated by H19 in the present ceRNA network. It has been reported that miR-29b-3p was regulated by H19 and promoted EMT in both CRC and bladder cancer [19, 20]. Two previous studies have shown that phosphorylation of key kinases in the PI3K/AKT/mTOR pathways was regulated by H19 through a ceRNA manner [21, 22]. Furthermore, H19 regulated many other cancer-related genes [23] in this network, such as AKT3, CSF1, MET, COL1A1, COL5A1, WWTR1, EPHB4, and TMPRSS3. Cox regression demonstrated that five of the mRNAs regulated by H19 are risk factors in CRC (Fig. 5). Furthermore, Kaplan–Meier analysis revealed that 11 of the mRNAs regulated by H19 are negatively correlated with the OS of CRC patients (Additional file 4).

We found that, in addition to H19, some other lncRNAs or pseudogenes in the present ceRNA network were also associated with OS or other important prognostic parameters. For example, lncRNA KCNQ1OT1 and the pseudogene RPLP0P2 predictors of poor survival in CRC. KCNQ1OT1 has been reported to associated with progression and metastasis of various cancers. Aberration of KCNQ1OT1 transcription was common in CRC. KCNQ1OT1 has been shown to mediate the growth of hepatocellular carcinoma by functioning as a ceRNA of miR-504 [24] and also to regulate proliferation and cisplatin resistance in tongue cancer via miR-211-5p-mediated Ezrin/Fak/Src signaling [25]. However, little is known about the function of the pseudogene RPLP0P2. By interacting with 15 miRNAs, RPLP0P2 regulates the expression of 50 mRNAs in the post-transcriptional level (Tables 1, 2). KEGG pathway enrichment analysis showed that these 50 mRNAs were enriched in the PI3K–Akt signaling pathway (FDR = 0.003) and pathways in cancer (FDR = 0.011). Thus, our results suggest that, similar to H19, RPLP0P2 may be an important oncogene and needs to be further studied.

A recent study has indicated that lncRNA interactions with miRNA and mRNA—such as H19, MALAT1, and KCNQ1OT1, HULC, and HOTAIR—could be potential diagnostic and prognostic biomarkers in cancer [26–28]. Some of these key lncRNAs were also found in the present ceRNA network. Since RPLP0P2, H19, and KCNQ1OT1 are associated with survival, they may serve as potential prognostic biomarkers for CRC.

This study has certain limitations. First, analysis of the ceRNA network was based on a bioinformatics algorithm using TCGA and published CLIP-seq data; no further experimental validation was performed. Several novel lncRNAs and pseudogenes with clinical significance in CRC need to be explored further to clarify the underlying molecular mechanism. Second, current research methods and theoretical systems of ceRNA are far from perfect. Research on lncRNA and pseudogenes is still developing, and many aspects need to be improved. Further experimental studies are needed to improve our understanding of the functional role of ncRNAs in CRC.

## Conclusions

In conclusion, the present study successfully applied bioinformatics analysis of large-scale samples in the TCGA database to identify cancer-specific lncRNAs and pseudogenes in CRC. The constructed ceRNA network, based on a large-scale CLIP-Seq data from the starBase database, provides a new approach to ncRNA research in CRC. It appears that H19 up-regulates various cancer-related mRNA expression level by serving as a ceRNA, and participates in the PI3K–Akt signaling pathway in this manner. Other ceRNAs like H19 in the network, such as pseudogene RPLP0P2, may also play a pivotal role in cancer progression by regulating the expression level of mRNAs through sponging various miRNA. The ceRNA network may help expand our comprehension of roles of transcriptomes, particularly non-coding transcripts, and improve our understanding of the pathogenesis of CRC and thus enable early diagnosis.

## Additional files

**Additional file 1.** The differentially expressed mRNAs, lncRNAs, pseudogenes, and miRNA in CRC.

**Additional file 2.** The miRNAs and their target mRNAs in the ceRNA network.

**Additional file 3.** GO terms of differentially expressed mRNAs in the ceRNA network.

**Additional file 4.** RNAs correlated with overall survival in the ceRNA network.

## Data Availability

The data of this manuscript can be download from The Cancer Genome Atlas database (https://portal.gdc.cancer.gov/).

## Abbreviations

CRC: colorectal cancer
ncRNAs: non-coding RNAs
lncRNA: long non-coding RNAs
EMT: epithelial–mesenchymal transition
ceRNA: competing endogenous RNA
miRNAs: microRNAs
MRE: miRNA response element
TCGA: The Cancer Genome Atlas
FDR: false discovery rate
FC: fold change
CLIP-seq: crosslinking-immunoprecipitation and high-throughput sequencing
GO: Gene Ontology
KEGG: Kyoto Encyclopedia of Genes and Genomes
ASOs: antisense oligonucleotides
NC: negative control
cDNA: complementary DNA
OS: overall survival
